# Late-Presenting Myocardial Infarction With PCI and Impella Placement Complicated by Ventricular Septal Defect and Impella Malfunction

**DOI:** 10.1177/23247096261454418

**Published:** 2026-05-20

**Authors:** Zaheen Hossain, Tai Metzger, Abdullah Jalal, James R. Burmeister, Ismail Zazay, Mohamed Abdalaziz, Tucker Billups

**Affiliations:** 1 159878 Department of Foundational Studies, Oakland University William Beaumont School of Medicine, Rochester, MI, USA; 2 Department of Medicine, John Sealy School of Medicine at UTMB, Galveston, TX, USA; 3 21818 Department of Internal Medicine, Corewell Health William Beaumont University Hospital, Royal Oak, MI, USA

**Keywords:** myocardial infarction, ventricular septal defect, impella, cardiogenic shock

## Abstract

Post–myocardial infarction ventricular septal defect (VSD) is a rare but frequently fatal mechanical complication. We report a 67-year-old man who presented approximately 48 hours after symptom onset with dyspnea and was diagnosed with an ST-segment elevation myocardial infarction. He underwent emergent percutaneous coronary intervention supported by Impella placement but subsequently developed a large ventricular septal defect two days later, complicated by Impella malfunction. The patient ultimately underwent successful surgical repair. This case highlights the importance of maintaining a high index of suspicion for mechanical complications in late-presenting myocardial infarction, even after apparent stabilization with revascularization and mechanical circulatory support.

## Introduction

Post–myocardial infarction ventricular septal defect (VSD) is an uncommon mechanical complication that carries a high risk of mortality. Its incidence has declined from approximately 1–2% in the pre–reperfusion era to around 0.3% with the widespread use of primary percutaneous coronary intervention (PCI).^1,2^ Despite improvements in early revascularization, outcomes remain poor, particularly in the absence of timely surgical intervention. VSDs most commonly occur between 4-14 days after an acute myocardial infarction (MI), typically in the context of delayed or unsuccessful reperfusion, and are often preceded by extensive myocardial necrosis.^3^ Established risk factors include advanced age, female sex, hypertension, and prolonged time to hospital presentation, all of which contribute to a higher likelihood of septal rupture.^4^

We present a rare case of a 67 year-old man who presented to the emergency department (ED) two days after onset of MI symptoms and developed a large post-MI VSD only two days after emergent PCI and Impella placement for an acute anterior ST-segment elevation myocardial infarction (STEMI). The case was further complicated by malfunction of the Impella device. This case highlights the need for heightened clinical suspicion in late-presenting MI patients and illustrates the challenges of managing post-MI VSD in the setting of mechanical circulatory support.

## Case Presentation

A 67 year-old male presented to the ED with a chief complaint of worsening shortness of breath (SOB) for the last two days. He had a past medical history of hypertension (HTN), hyperlipidemia (HLD), tobacco use, chronic kidney disease (CKD), and type II diabetes mellitus (T2DM). He recently traveled to the U.S from his home country.

On the physical exam the patient was alert, oriented, and in no acute distress. Cardiac exam showed regular rate and rhythm, normal S1 and S2, 3/6 pansystolic murmur at the left sternal border. The rest of the exam was unremarkable. A 12 lead electrocardiogram (EKG) showed significant ST-segment elevations in inferior, anterior, and lateral leads. Troponin I levels were 11.27. Echocardiogram initially showed the apical cap, apical septal wall, apical anterior, and apical inferior wall as akinetic. The mid anteroseptum was hypokinetic. The left ventricular ejection fraction was estimated at 35%. A large thrombus was noted present at the apex of the left ventricle measuring 1.62 X 2.53 cm. VSD however, was not initially present.

The patient received aspirin 324 mg and was started on a heparin infusion and nicardipine infusion. Propofol was used for sedation, with triglyceride monitoring. The following day, aspirin 81 mg daily, prasugrel 10 mg daily, and atorvastatin 80 mg nightly were initiated.

**Figure 1.**
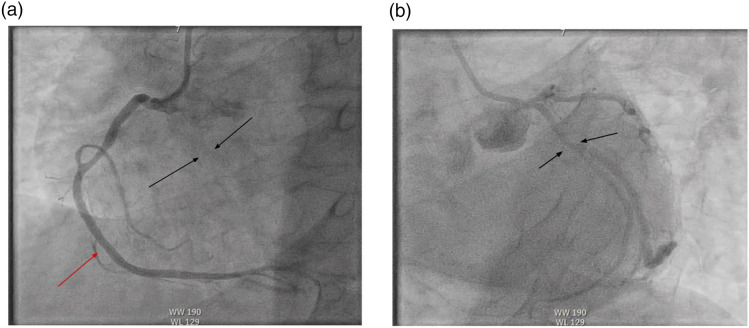
(A) Coronary angiography demonstrating left anterior descending artery (LAD) occlusion; the black arrows indicate the expected course of the LAD, and the red arrow indicates the right coronary artery (RCA). (B) Restoration of LAD flow after PCI, indicated by the black arrows.

He underwent PCI of the left anterior descending artery (LAD) into the left main (Figure 1). He had marked decrease in ejection fraction (EF) with left-sided Impella placement with improvement in his hemodynamics. The patient was intubated and transferred to the cardiac intensive care unit with Impella support. The hospital course was further complicated by cardiogenic shock, acute kidney injury (AKI) requiring continuous renal replacement therapy, colitis, pulseless electrical activity (PEA) cardiac arrest, atrial fibrillation with rapid ventricular response, and subsequent complete heart block requiring transvenous pacing.

**Figure 2.**
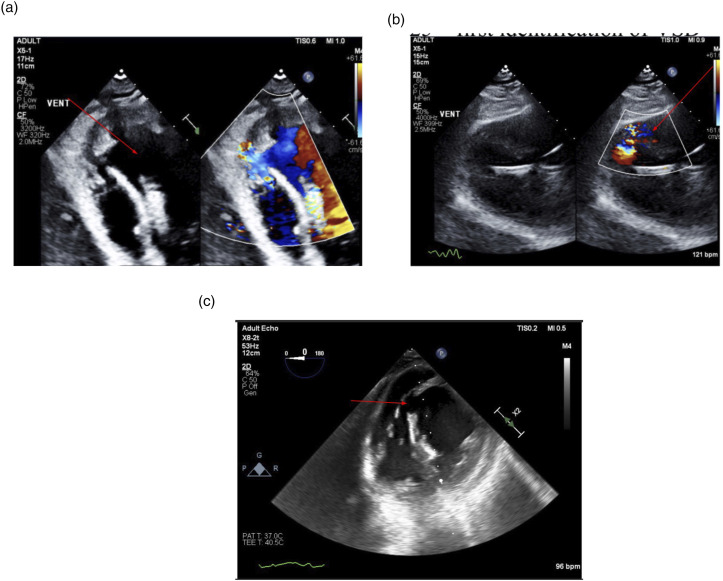
(A–C) Echocardiography demonstrated a large muscular ventricular septal defect, indicated by red arrows, with left-to-right shunting on color Doppler. A left ventricular apical thrombus was also present.

2 days after the PCI, the patient’s cardiac output suddenly increased on the Impella from 7.6 L/min to 13.9 L/min. Initially, it was believed that the patient’s cardiac function was recovering. However, the patient’s blood pressure started dropping evidenced by the SVR decreasing from 1074 mmHg/L/min to 426 mmHg/L/min and the mixed venous oxygen saturation increased from 79.2% to 84.8%. These signs all hinted towards a VSD. As a result, a bedside transthoracic echocardiogram (TTE) was performed showing a new VSD and LV thrombus (Figure 2). TTE demonstrated a large muscular VSD measuring 1.66 cm with a peak gradient of 45 mmHg, as well as a left ventricular apical thrombus measuring 1.93 × 1.00 cm. Approximately sixteen days later, while waiting for VSD repair surgery, the motor of Impella CP stopped functioning. A 5.5 Impella was emergently inserted from the right axillary artery.

VSD repair with a bovine pericardial patch was completed three days after the second Impella was emergently placed. The Impella was removed three days after VSD repair and there was only residual VSD. On three month follow-up the patient had improved from a cardiovascular standpoint, with the EF improved from 35% after the STEMI to 45% at this visit.

## Discussion

This is a rare case in which a VSD occurred after the placement of an Impella following a late presenting MI. VSD following STEMI only occurs 1-2% of the time. When they occur, they classically develop 4–14 days after MI, particularly in the setting of delayed or unsuccessful reperfusion. VSDs result in an abnormal communication between the left and right ventricles, resulting in a left-to-right shunt. During systole, blood from the higher-pressure left ventricle is ejected not only into the aorta, but also across the VSD into the right ventricle. This increases blood flow into the right ventricle and subsequently into the pulmonary circulation. This increase in pulmonary artery blood flow (Qp) relative to the systemic circulation (Qs) leads to a marked increase in pre-load of the pulmonary circulation and the left heart chambers. In the early phase, higher systemic compared to pulmonary vascular resistance favors a predominantly left-to-right shunt, which augments cardiac output by increasing the volume of blood recirculated through the lungs and heart. This is seen by an increase in left ventricular end-diastolic volume and stroke volume, as well as increased right ventricular output proportional to the size of the shunt. Over time, if pulmonary vascular resistance rises, the shunt may decrease or reverse, but in the early phase the net effect is an increase in cardiac output due to the recirculation of oxygenated blood.

In this case, a PCI was performed, and Impella support was initiated shortly after STEMI diagnosis, as revascularization is crucial to avoid cardiac muscle necrosis, but the VSD still occurred 2 days following the STEMI. Our case shows the importance of recognizing post-MI complications even when they occur outside of the timeframes described in the literature. Another key learning point is the fact that increased cardiac output doesn’t necessarily mean that cardiac function has improved. In this case, the increased cardiac output following Impella placement was due to the VSD shunting blood from left ventricle to right ventricle, increasing preload of the left ventricle. This case also emphasizes the increased likelihood of MI complications in patients who present multiple days after symptom onset.

In one study, the median time to hospital arrival was four hours, with an interquartile range of two to seven and a half hours,^5^ whereas our patient presented approximately 48 hours after symptoms began. To our knowledge, prior reports specifically describing post-PCI VSD in a late-presenting MI complicated by Impella malfunction are limited.

While there has been relatively little research into the incidence of VSD in patients presenting late with MI, hospital outcomes of patients who present late with MI is well studied. One retrospective study examined 275 patients with ST-segment elevation myocardial infarction (STEMI) who presented late (12–72 hours after symptom onset) and underwent PCI. 31% experienced a composite outcome of death, recurrent infarction, or heart failure hospitalization over a median follow-up of 61 months, with higher rates in those with initial Killip class ≥2. Outcomes were worse among patients who were older, had reduced ejection fraction, higher Killip class, or multivessel disease. Patients presenting 12–48 hours after onset had better long-term outcomes compared to those presenting 48–72 hours after onset. The study concluded that earlier intervention within the late presentation window and careful risk stratification may improve prognosis in this high-risk group.^6^

There has also been significant research into the use of Impella in the context of post MI VSD. A systematic review by Gemelli et al. evaluated 20 studies encompassing 68 patients who received Impella support before treatment of post-myocardial infarction ventricular septal defect (post-AMI VSD), most of whom were in cardiogenic shock. The majority had posterior VSDs, and 72% underwent surgical or percutaneous closure, with an overall in-hospital mortality of 47%, with lower mortality observed in patients supported with surgically Impella devices compared with percutaneous systems (35% vs 58%)). Impella provided effective left ventricular unloading, improved peripheral perfusion, and reduced left-to-right shunting, sometimes in combination with VA-ECMO (“ECMELLA”) for biventricular support. However, complications occurred in 43% of patients, most commonly major bleeding, hemolysis, and limb ischemia; with higher rates in percutaneous devices. The findings suggested that Impella can be a valuable bridge-to-repair strategy in unstable post-AMI VSD patients, but careful device selection, monitoring, and management are essential to balance benefits with risks.^7^

Although Impella has been increasingly studied in the management of post–MI VSD, we could not identify any cases of VSD occurring in a late-presenting patient after PCI following malfunction of Impella. A case series by Jalli et al. reported on 13 patients with cardiogenic shock from post-infarction ventricular septal defect (PIVSD) who received Impella support at two centers between 2016 and 2022. Most patients received an axillary Impella 5.0 or 5.5, with median implantation five days after MI, and median support lasting about 12 days. Seven patients underwent successful VSD closure (six surgical, one percutaneous) with all surviving to 30 days, while overall 30-day mortality was 46%, primarily from multi-organ failure. Survivors tended to be younger, diagnosed and supported earlier, underscoring that prompt recognition and early mechanical circulatory support may improve survival to closure in this high-risk condition.^8^

In a related case report, a 63-year-old woman presented late with anterior STEMI and cardiogenic shock, later found to have a large post-MI VSD with a significant left-to-right shunt. Initial stabilization with an intra-aortic balloon pump failed, leading to further deterioration. An Impella CP® device was inserted on day seven post-MI, producing rapid hemodynamic improvement and enabling withdrawal of other supports. She underwent urgent cardiac transplantation five days later, recovering with only mild residual deficit from a postoperative stroke, highlighting Impella’s potential as a bridge to definitive therapy in refractory cardiogenic shock.^9^ Similarly, Shezad et al. described a 58-year-old man with an anterior myocardial infarction complicated by an apical ventricular septal defect, managed initially with Impella CP® support before angioplasty and later upgraded to an axillary Impella 5.5®.^10^ Surgery was delayed for 28 days to allow myocardial scarring, during which the patient underwent physical therapy to maintain conditioning. He subsequently had a successful surgical VSD repair with uneventful recovery, highlighting the value of prolonged mechanical support and multidisciplinary planning in optimizing outcomes.

This case illustrates an uncommon presentation of post–MI VSD occurring just two days after emergent PCI and Impella placement, complicated by device malfunction, in a patient with a late-presenting anterior STEMI. The atypical timing emphasizes that mechanical complications can arise unexpectedly, particularly in patients with prolonged symptom onset before reperfusion. Prompt recognition and surgical intervention were critical to survival, highlighting the importance of ongoing hemodynamic monitoring and diagnostic vigilance even after apparent stabilization. This case also underscores the need for individualized management strategies when mechanical circulatory support devices are involved, as device failure can significantly alter the clinical trajectory.
